# Effect of Peptides on the Synthesis, Properties and Wound Healing Capacity of Silver Nanoparticles

**DOI:** 10.3390/pharmaceutics15102471

**Published:** 2023-10-16

**Authors:** Afroditi Papaioannou, Angeliki Liakopoulou, Dimitris Papoulis, Eleni Gianni, Patroula Gkolfi, Eleni Zygouri, Sophia Letsiou, Sophia Hatziantoniou

**Affiliations:** 1 Department of Pharmacy, University of Patras, 26504 Patras, Greece; afropolpapa@gmail.com (A.P.); aggelikiliakopoulou27@gmail.com (A.L.); 2 Department of Geology, University of Patras, 26504 Patras, Greece; papoulis@upatras.gr (D.P.); elengian93@gmail.com (E.G.); 3 Chemical Process & Energy Resources Institute, Centre for Research & Technology Hellas (CERTH), 15125 Athens, Greece; 4 Department of Chemistry, University of Patras, 26504 Patras, Greece; patroula.gkolfi@gmail.com (P.G.); eleni0503zig@hotmail.com (E.Z.); 5 Department of Food Science and Technology, University of West Attica, Agiou Spyridonos 28, 12243 Aegaleo, Greece; sletsiou@gmail.com

**Keywords:** AgNP, silver nanoparticles, peptide conjugates, wound healing, scratch test, myristoyl tetrapeptide 6, copper tripeptide 1

## Abstract

The aim of this study is the synthesis of novel peptide–silver nanoparticle conjugates with enhanced wound healing capacity. Peptide–silver nanoparticle conjugates were synthesized using myristoyl tetrapeptide 6 (MT6) or copper tripeptide 1 (CuTP1). Peptide-free silver nanoparticles (AgNP) were synthesized using NaBH4 and sodium citrate and were used as control. The addition of the peptides during or after the synthesis of nanoparticles and its impact on the properties of the synthesized peptide–silver nanoparticle conjugates were assessed. The monitoring of the synthesis of nanoparticles was achieved using ultraviolet–visible spectrophotometry (UV–/Vis). The characteristics and colloidal stability of the nanoparticles (size and ζ-potential distribution, morphology, composition and structure) were monitored using dynamic laser scattering (DLS), transmission electron microscopy (TEM), atomic absorption spectroscopy (AAS) and X-ray diffraction (XRD). The wound healing capacity of the peptide–silver nanoparticle conjugates was assessed using scratch test assay on fibroblasts (NIH/3T3). The results indicated that the addition of the peptides during the synthesis of nanoparticles lead to better yield of the reaction and more effective capping while the size distribution and ζ-potential of the conjugates indicated long-term colloidal stability. The MT6-AgNP conjugate exhibited 71.97 ± 4.35% wound closure, which was about 5.48-fold higher (*p* < 0.05) than the corresponding free MT6. The CuTP1-AgNP conjugate exhibited 62.37 ± 18.33% wound closure that was better by 2.82 fold (*p* < 0.05) compared to the corresponding free CuTP1. Both peptides led to the synthesis of silver nanoparticle conjugates with enhanced wound healing capacity compared to the respective free peptide or to the peptide-free AgNP (29.53 ± 4.71% wound closure, *p* < 0.05). Our findings demonstrated that the synthetized peptide–silver nanoparticle conjugates are promising ingredients for wound care formulation.

## 1. Introduction

Nanotechnology is a rapidly growing scientific field with applications across various industries. The properties of nanoparticles differ from ingredients with conventionally sized particles, and this is mainly due to their high surface area/volume ratio, improved substance transport capacity and higher reactivity. Additionally, modification of their surface properties is an easy process, making them versatile for use in many applications [1,2]. The application of nanomaterials in the field of biomaterials and in particular in pharmaceutical products has recently been of great interest. Specifically, silver nanoparticles are used as adjuvants in vaccines, antidiabetic agents, biosensors, in wound healing and in anticancer therapy [3,4]. Additionally, nanoparticles can be tethered with differently functionalized entities that enhance their efficacy in drug delivery and therapeutic response [5]. Several biologically active molecules have been conjugated with silver nanoparticles so far, taking advantage of this unique feature rendering them as the “added-value” materials as they possess the properties from both the moieties in-connection [6]. Peptides are a promising class of biologically active molecules that have been conjugated with silver nanoparticles. The use of peptides has found applications in many areas of health as they demonstrate a remarkable effect. They have also been linked to other materials such as nanoparticles and antibiotics to enhance their action against bacteria [7,8].

The conjugation of peptides with silver nanoparticles possesses enhanced targeting capability and activity while its toxicity is diminished, traits that may be attributed to the peptides as nanoparticles do not possess them prior to conjugation [9,10].

Nanotechnology can be applied with great success in accelerating various phases of the healing process of both acute and chronic wounds. Topical delivery of drugs and antimicrobial agents for wound healing has been improved by combining them with nanomaterials, nanoscaffolds, nanofibers and biomaterials that are incorporated into silver nanoparticles [11,12].

Thus, advanced conjugates of peptides on silver nanoparticles were developed by several researchers for their applications in different fields. These nanocomposites had a synergetic effect on their initial components, combined with avoidance of aggregation of nanoparticles, low level of hemolytic activity, low toxicity, bioavailability, high specificity and easy tailoring [13,14,15,16]. Until now, such nanocomposites had been tested for their antibacterial applications [17,18,19] as stabilized agents [20] and wound healing applications [21,22]. Silver nanoparticles’ importance in wound healing has been underlined by Paladini et al. in 2019 [11]. Additionally, it has been claimed that AgNPs accelerate the wound-healing process by enhancing the anti-inflammatory properties, by increasing the antibacterial activity against a vast number of bacterial strains, by being incorporated in wound dressings and [5] by altering the surface of the wound, facilitating drug delivery [23,24].

The synthesis of silver nanoparticles with LL37 peptide was performed using photochemical synthesis, as an antimicrobial and anti-biofilm agent aiming to promote wound healing and prevent possible infections in burn care [21]. Moreover, a silver-impregnated biomaterial combined with an ultrashort peptide (Ac-LIVAGK-NH_2_) under UV-light was investigated for its antimicrobial efficiency in antibiotic-resistant *P. aeruginosa* bacteria aiming to be used in wound healing applications after surgeries or chronic and large surface wound treatment mainly on diabetic ulcers and severe burns [20]. Myristoyl tetrapeptide-6 (MT6) is a biomimetic peptide with the amino acid sequence H-Val-Pro-Ala-Ala-NH2 (L-valyl-L-prolyl-L-alanyl-L-alaninamide). It shows affinity for Transforming Growth Factor beta (TGF-β) receptors and thus induces production of type I procollagen and fibronectin. Because of this characteristic, it is used as a cosmetic ingredient and its main claim is protection against environmental aging factors such as UV radiation. It exhibits anti-aging and anti-wrinkle effect and is used for protection against environmental stimulus such as UV irradiation on anti-wrinkle, skin re-generation, skin lifting and firming cosmetics [25].

Copper tripeptide-1 (CuTP1) is a complex of the tripeptide L-Glycyl-L-Histidyl-L-Lysine with copper. It is a cosmetic ingredient with multiple claims of action. It is considered to increase the production of essential skin proteins, such as collagen and fibronectin, as well as other important components of the skin’s extracellular matrix, while at the same time, it is characterized as an agent for restoring the function of irradiated fibroblasts. It can be incorporated into anti-wrinkle cosmetics and formulations for reconstruction of the skin as it seems to favor the reduction of deep lines and wrinkles on the forehead and around the eye area. In addition, it is used on the scalp for hair care.

CuTP1 was first isolated from human plasma in 1973, and its wound healing properties were reported in 1988 by Maquart and colleagues. They concluded that GHK and its complex with copper acted as activators of tissue regeneration [26,27].

This tripeptide is part of collagen and is produced in large quantities by injured tissues, having a dual behavior, as it acts as a Cu transporter peptide and as a signaling peptide. GHK spontaneously combines with copper, creating the GHK-Cu complex and facilitates the stabilization, transport and absorption of copper in cells. Through interaction with growth factors, it promotes the in vivo synthesis of many necessary rebuilding components (glycosaminoglycans, proteoglycans, elastin and collagen), hair growth, while increasing the rate of growth and migration of different types of cells involved in anti-inflammatory and antioxidant responses [27,28]. 

Many enzymes essential to the health and appearance of the skin, such as lysyl oxidase, which is essential in the production of collagen and elastin, superoxide dismutase, an antioxidant, tyrosinase and cytochrome-c oxidase require copper as a cofactor. Also, GHK-Cu regulates MMPs and their inhibitors, while reducing collagenase activity [29,30,31,32].

As a cosmetic ingredient, the CuTP1 complex is considered to improve skin firmness and texture. Furthermore, the above mechanisms justify its ability to increase the elasticity of the skin, as well as to reduce the depth of wrinkles, signs of photoaging and dark spots. Finally, the CuTP1 complex is effective in tissue reconstruction, angiogenesis and wound healing [32]. The present study focuses on the synthesis and characterization of peptides conjugated with silver nanoparticles to explore their effect on the skin-related wound healing process. The conjugation of two peptides MT6 and CuTP1 with silver nanoparticles were investigated for the first time to our knowledge, and their ability to enhance cell migration was tested in vitro in wound treatment applications.

## 2. Materials and Methods

### 2.1. Materials

All the chemicals used were of analytical grade. Silver nitrate (Fisher Scientific, Leicester, UK), water for injection (WFI) (Demo S.A., Pharmaceutical Industry, Kryoneri, Attica, Greece), methanol (Fisher Scientific, Leicester, UK), NaBH_4_ (Fluka chemie GmbH, Buchs, Switzerland), sodium citrate (Sigma-Aldrich, Darmstadt, Germany) and phosphate buffered saline (PBS) (Sigma-Aldrich, Darmstadt, Germany) were purchased. DermaPep™A420 (MT6, INCI: myristoyl tetrapeptide 6) and DermaPep^®^ CuP (CuTP1, INCI: copper tripeptide 1) both from BeadTech Inc., Gyeonggi-do, Republic of Korea, were commercially available cosmetic grade ingredients and were donated by Cosmochem chemicals S.A., Athens, Greece.

### 2.2. Synthesis of Silver Nanoparticles

Silver nanoparticles were prepared by adding either the MT6 or CuTP1 peptide i. during AgNO_3_ reduction (MT6-AgNP, CuTP1-AgNP) or ii. after synthesis of nanoparticles (AgNP MT6, AgNP CuTP1). Silver nanoparticles were also synthesized without peptide interaction (AgNP) and were used as control.

The synthesis of AgNP was achieved according to Siakavella et al. with modifications using NaBH_4_ as a reducing agent and sodium citrate as a stabilizer (capping agent) [32]. Initially, 20 mM NaBH_4_ and 0.6 mM sodium citrate solutions in 1:1 (*v*/*v*) ratio were mixed under stirring (300 rpm, 30 min) in an ice bath. A 10 mM AgNO_3_ solution was added to the mixture dropwise in a 1.25:2 (*v*/*v*) ratio. After leaving the mixture undisturbed for 24 h, it was centrifuged (13,000 rpm, 15 °C, 15 min) and the sediment was collected. The procedure was repeated twice, and the combined sediments were stored at 4 °C after dispersing it in 10 mL WFI.

For the synthesis of the peptide–AgNP conjugate, each peptide was diluted in WFI to a final concentration of 250 ppm for MT6 and 125 ppm for CuTP1.

MT6-AgNP and CuTP1-AgNP were synthesized by mixing each peptide solution with the 10 mM AgNO_3_ solution in 1:1 (*v*/*v*) ratio. This mixture was added to NaBH4 and sodium citrate solution in a 2.5:2 (*v*/*v*) ratio. Purification and recovery of the nanoparticles were performed as described [33].

AgNP MT6 and AgNP CuTP1 were synthesized by adding each peptide dispersed in WFI to the synthesized AgNP dispersion in a 1:1:3 (*v*/*v*) ratio of peptide/WFI/AgNP, respectively.

### 2.3. Monitoring of Silver Nanoparticle Synthesis Using Spectrophotometry

The successful synthesis of silver nanoparticles of each sample was affirmed by monitoring the presence of an absorption peak at around 400 nm using a UV-1800 UV–vis spectrophotometer (SHIMADZU, Kyoto, Japan) [34,35]. The samples were placed in quartz cuvettes and were diluted with deionized water, adjusting their absorption below or near 1.

### 2.4. Particle Size and ζ-Potential Characterization and Stability Study of Silver Nanoparticles

The mean size (average hydrodynamic diameter) and ζ-potential of nanoparticles were monitored using dynamic light scattering (DLS) and electrophoretic light scattering (ELS), respectively (Zetasizer, Nano ZS, Malvern Panalytical, Malvern, UK), equipped with a He–Ne Laser beam at a fixed backscattering angle of 173°. The samples were diluted in WFI (sample/WFI 1:6 *v*/*v*). Measurements for each sample as the average of 100 runs with the phase analysis light scattering mode, after equilibration at 25 °C, were repeated three times at 25 °C. The ζ-potential was calculated according to the Smoluchowski theory.

The nanoparticle dispersions were stored at 4 °C and their colloidal stability were assessed for a period of three months by monitoring the mean size and ζ-potential of their particles, at predetermined time intervals (1, 15, 30, 60 and 90 days after preparation).

### 2.5. Morphology of Silver Nanoparticles

The morphology and the size of the nanoparticles were observed using a transmission electron microscope (TEM, JEOL: JEM-2100, JEOL Ltd., Tokyo, Japan) after placing a drop of each dispersion on a copper grid (200 ms), covering it with a thin film of carbon and allowing to stand at room temperature until the water phase evaporated completely. The selected area electron diffraction (SAED) patterns that certify the crystallinity of the nanoparticles were also studied. The graphs of silver nanoparticles’ size distribution were constructed after analyzing TEM images using ImageJ [36].

### 2.6. Quantification of Ag in Silver Nanoparticles Using Atomic Absorption Spectroscopy (AAS)

The quantification of Ag in the synthesized nanoparticles was achieved using graphite furnace atomic absorption spectroscopy (AAS) (Analyst 300, Perkin-Elmer, Waltham, MA, USA) [37]. The dispersions were diluted with deionized water, so that the Ag concentration was within the detection limits of the instrument (ppb), and the concentration of Ag in the initial samples was calculated taking into account the dilutions carried out each time.

The yield of each synthesis method was calculated considering the initial (Ci) and the final (Cf) concentrations of Ag in the dispersions of silver nanoparticles, according to Equation (1).

(1) $$\mathrm{yield}\;\left(\%\right)=\frac{\mathrm{Cf}}{\mathrm{Ci}}\times 100$$

where Cf is the Ag concentration measured by AAS and Ci is the initial Ag concentration corresponding to the volume of 10 mM AgNO_3_ solution used for each sample. 

### 2.7. Structural Characterization of Silver Nanoparticles Using XRD

The structural characterization of nanoparticles was determined using X-ray diffraction with *λ* = 1.5418 Å Cu-K_α_ radiation and nickel filter (Bruker D8 Advance Diffractometer, Berlin, Germany) using Si holders for the nanoparticles. The samples were first lyophilized, as they were required to be in solid phase, so that they could later be dispersed in ethanol. Each sample, after treatment with ethanol, was placed in the center of the Si sample holder and the diffraction spectra were taken.

For the calculation of silver particle size of the samples, the Scherrer formula (Equation (2)) was used.

(2) $$D=\frac{K\times \lambda }{\beta_{\mathrm{hkl}}\times \cos\theta_{\mathrm{hkl}}}$$

where D is the crystallite size, K is a numerical constant, which is 0.93 as it was derived by Patterson et al. in 1939, *λ* is the XRD wavelength, which is equal to 0.154 nm, *β*_hkl_ is the π/180 × full width half maximum (FWHM) and *θ*_hkl_ is the Bragg-diffraction angle. For the silver particles, the characteristic peak of 38.13° of XRD was used [38].

### 2.8. Cell Culture

The cell line that was used was mouse embryo fibroblast (NIH/3T3, ATCC, Manassas, VA, USA, #CRL-1658) that was cultured at 37 °C in a humid 5% CO_2_ incubator (Thermo Scientific Forma Series II, Karlsruhe, Germany) and grown in Dulbecco’s modified Eagle’s medium (DMEM, Pan-biotech, Aidenbach, Germany), containing 4.5 g/L glucose, 110 mg/L sodium pyruvate and L-glutamine, supplemented with 0.1% penicillin/streptomycin (10.000 U:10 mg/mL, Biosera, Nuaille, France), 250 μg/mL sterile filtered 1:100 amphotericin B solution (Sigma-Aldrich, Taufkirchen, Germany) and 10% FBS (PanReak Applichem Darmstadt, Germany). To arrest proliferation, the cells were kept in DMEM free of FBS 48 h prior to the in vitro scratch assays. 

### 2.9. Scratch Assays

The cells were placed in 24-well tissue culture plates (Sarstedt, Nümbrecht, Germany) and maintained until they were 95% to 100% confluent. Consequently, a scratch was created across each well by holding vertically a plastic cell scratcher of 1 mm width (SPLScar Scratcher, SPL Life Science Co., Gyeonggi-do, Republic of Korea). The detached cells were then removed by washing each well twice with Dulbecco’s phosphate buffered saline without Ca^2+^ or Mg^2+^. Fresh DMEM growth medium free of FBS was replenished in each well. Images of the initial scar (t = 0) were obtained using a microcamera (Soft Plus, Callegari, Parma, Italy) at 400× magnification and 640 × 480 resolution. Test solutions or controls were added in each well and cell migration (scratch closure) was monitored for a period of 48 h, photographing each scratch at 24 h and 48 h. DMEM supplemented with 2% FBS was used as positive control (control+). DMEM supplemented with 0.1% bovine serum albumin (BSAPanbiotech, Aidenbach, Germany) as well as the solvent of the samples was used and as negative control (control−). The images acquired for each sample were further analyzed quantitatively by using ImageJ [36]. For each image, the wound area was calculated after measuring the length and the distances between the sides of the scratch. Wound closure was determined at 24 and 48 h according to Equation (3).

(3) $$\left(\%\right)\mathrm{Wound}\;\mathrm{Closure}=\frac{A_{t0}-A_{\Delta t}}{A_{t0}}\times 100$$

where A_t0_ is the wound area at time 0 and A*_Δ_*_t_ is the wound area after 24 h or 48 h of the initial scratch both in mm [2,39,40].

### 2.10. Statistical Analysis

The statistical significance of differences was estimated by Student’s *t*-test (Microsoft Office Excel 2007, Redmond, WA, USA) at a 95% level of significance. The results are reported as mean values ± standard deviation (SD) of measurements executed in triplicate where possible.

## 3. Results

### 3.1. Synthesis of the Silver Nanoparticles

The efficient preparation of silver nanoparticles and their presence in the samples were verified by UV–vis spectrophotometry. All the synthesized nanoparticles presented a peak maximum at the range of 397 nm to 453 nm (Table 1), indicating the successful synthesis of silver nanoparticles following either method tested [34,35].

### 3.2. Ag Quantification and Synthesis Method Yield

The Ag concentration in the samples and the synthesis method yield was calculated using Equation (1) based on results acquired using AAS. 

The measured Ag concentration in the samples ranged from 10.78 ± 1.52 ppm for AgNP CuTP1 to 363.88 ppm ± 0.97 ppm for AgNP, rendering a calculated yield from 13.14 ± 2.01% to 88.68 ± 0.20%, respectively (Table 2).

The addition of peptides during the synthesis of nanoparticles (MT6-AgNP and CuTP1-AgNP) had higher yields (*p* < 0.05) compared to their respective AgNP MT6 and AgNP CuTP1. The conjugates of MT6 had better yield compared to the corresponding CuTP1 conjugates (0.05), indicating a role of the peptide type in the procedure. 

### 3.3. Size and ζ-Potential and Stability of Silver Nanoparticles

The average hydrodynamic diameters (nm), the size polydispersity indices (PdI) and the ζ-potential values of the nanocomposites are summarized in Table 2. 

The peptide–AgNP conjugates possessed a higher hydrodynamic diameter (from 91.430 ± 0.658 nm to 181.600 ± 3.554 nm) in comparison to conventional AgNP (60.440 ± 0.964 nm), possibly due to the presence of peptide molecules on their surfaces. 

MT6-AgNP and CuTP1-AgNP had much higher hydrodynamic diameters (*p* < 0.005) than their respective AgNP MT6 and AgNP CuTP1.

All the tested samples had a PdI ranging from 0.375 ± 0.017 (CuTP1-AgNP) to 0.510 ± 0.010 (AgNP). 

The average crystallite size of the nanoparticles calculated using the Scherrer formula (Equation (2)) was based on the most intense peak (111) in the XRD diffractograms. The results are summarized in Table 3. These results are not close with the results obtained using either DLS or TEM images. Τhis discrepancy may be due to the structure of conjugates that may have a “bunch of grapes” structure, which consists of small silver nanoparticles surrounded by peptides, or due to the limitations of the use of Scherrer formula, which produces more accurate results for spherical nanoparticles [41].

The absolute values of ζ-potential ranged from below 15 mV for AgNP-MT6 and AgNP-CuTP1 to above 20 mV for MT6-AgNP and AgNP-CuTP1, indicating that the synthesis procedure may affect the colloidal stability of the peptide–AgNP conjugate (Table 2).

**Figure 1:**
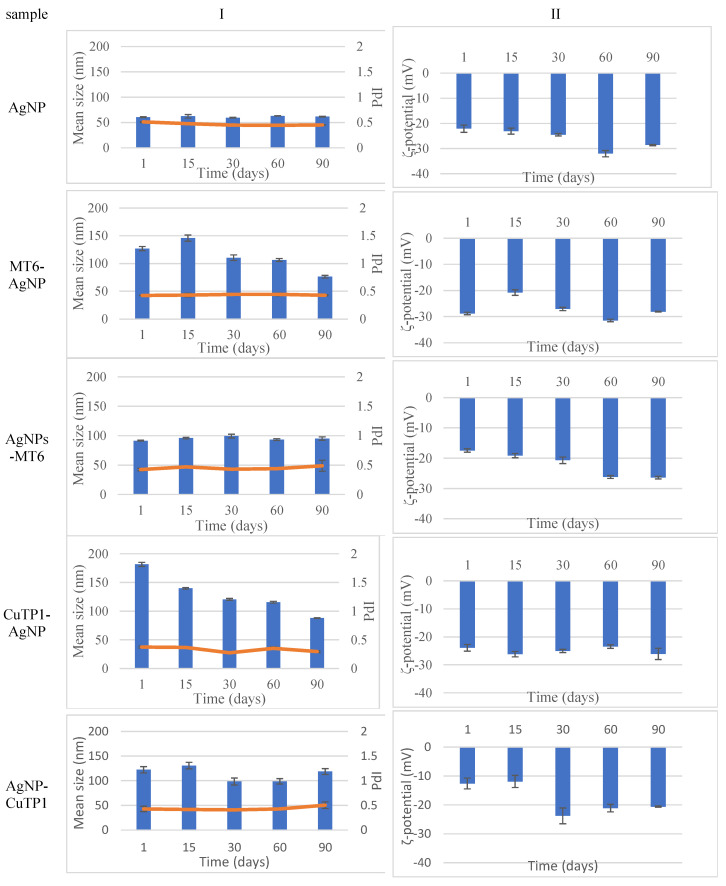
Stability study monitoring mean size (**I**) and ζ-potential (**II**) of silver nanoparticles for 90 days.

The stability study of the dispersions of all nanoparticles (Figure 1) revealed that at the end of a period of 90 days, the size of peptide–AgNP conjugates was adjusted around 100 nm, while conventional AgNP was around 50 nm. The absolute value of the ζ-potential of all nanoparticles was over 20 mV, indicating a satisfactory long-term stability.

### 3.4. Morphology of Silver Nanoparticles

**Figure 2:**
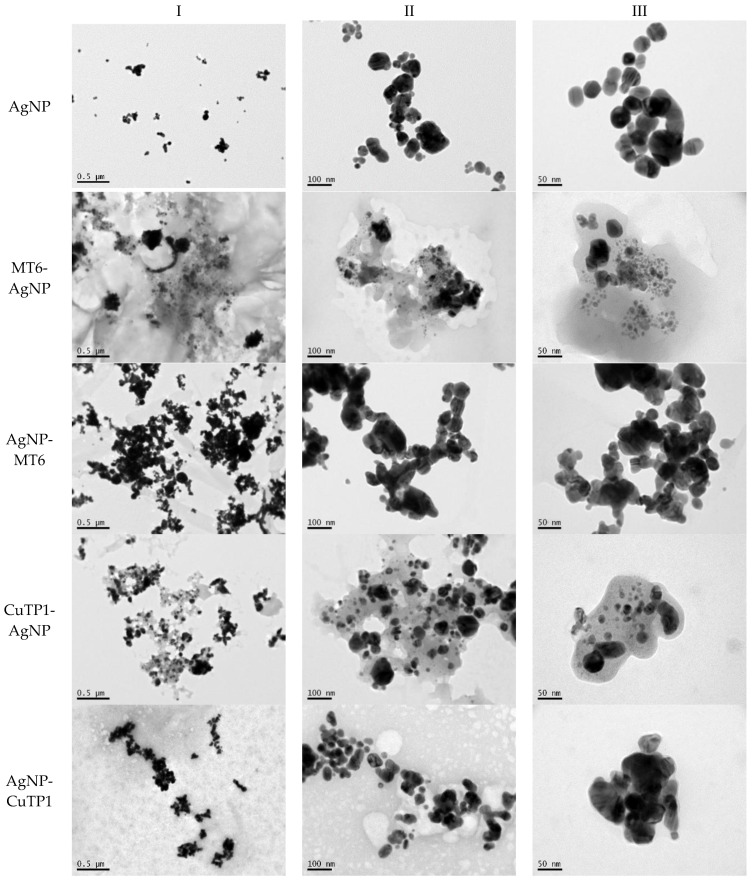
TEM images of silver nanoparticles observed under 4000 (**I**), 15,000 (**II**) and 300,000 (**III**) magnitude. Bar represents 500 nm (**I**), 100 nm (**II**) and 50 nm (**III**).

TEM images verified the presence of spherical or almost spherical shaped nanoparticles (Figure 2) [42]. A dense halo around MT6-AgNP and CuTP1-AgNP was observed, probably attributed to the presence of peptides on the surface of nanoparticles. This halo was not observed in conventionally synthesized AgNP [32]. The particle size distribution was calculated using image analysis and was similar for all samples (Table 4). AgNP-MT6 and AgNP-CuTP1 formed smaller dense aggregates without a noticeable halo.

### 3.5. Crystallinity of Silver Nanoparticles

**Figure 3:**
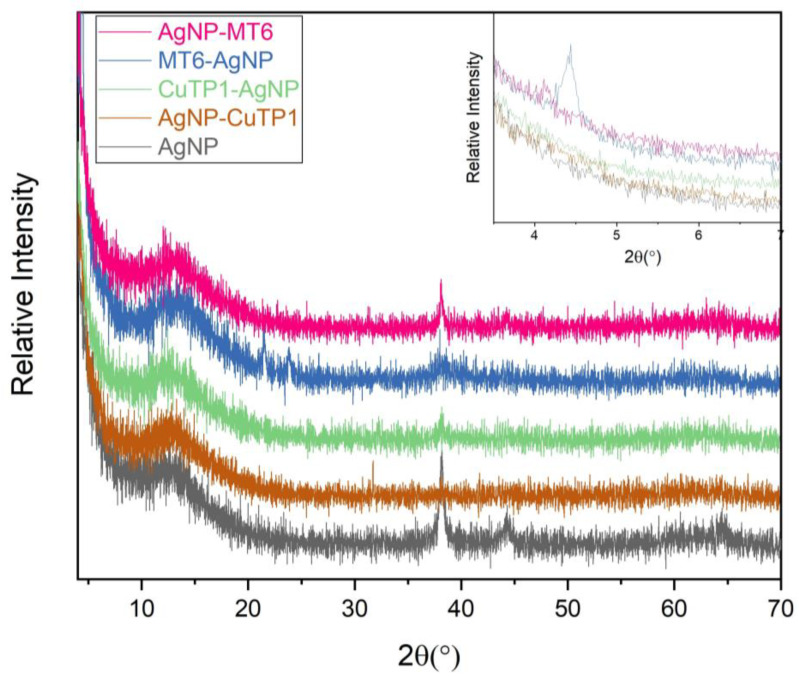
XRD patterns of silver nanoparticles.

The SAED patterns that are summarized in Table 4 revealed that the distances from the diffraction centers for all the synthesized nanoparticles were similar. This result indicates the similar crystallinity of the samples in agreement with their XRD spectra (Figure 3).

The characteristic peak of silver nanoparticles was observed at around 38.13°, corresponding to (111) plane, which is in agreement with the available literature [32,43]. The peak is not intense in AgNP-CuTP1 due to the small percentage of AgNP in the composite (13.14 ± 2.01% yield), as it was calculated also using UV–vis spectrophotometry (see Table 1). In the case of pure AgNP, the characteristic peak of silver at 44.32° is also visible. The XRD patterns of peptide–AgNP conjugates (MT6-AgNP, AgNP-MT6, CuTP1-AgNP, AgNP-CuTP1) do not present such intense peaks of silver due to the interaction of silver particles with organic-nature materials. The broad peak between 10° and 20° 2θ is characteristic for the pure crystalline phase, almost amorphous of NaBH_4_ structure, that was used for the nanocomposite synthesis. This is in agreement with the available literature for the investigation of pure phase of NaBH_4_. The sharp peaks of NaBH_4_ are missing due to the lack of high crystallinity in the remaining NaBH_4_ [44]. The peaks at about 21° and 24° 2θ in the sample MT6-AgNP are an indication of the remaining particles of AgNO_3_ after the synthesis procedure. These peaks frequently appeared in XRD patterns of silver nanoparticles where AgNO_3_ was used [45]. The presence of new peaks at small angles is observed in the XRD patterns of MT6-AgNP and AgNP-MT6, while it is more intense in the case of MT6-AgNP. This result may be explained by the difference in the structure of MT6-AgNP compared to the other synthesized nanoparticles as was revealed from SAED patterns. The presence of an intense peak at 4.44° may reflect this structural variation. 

### 3.6. Cell Migration Rate—Wound Healing Capacity

**Figure 4:**
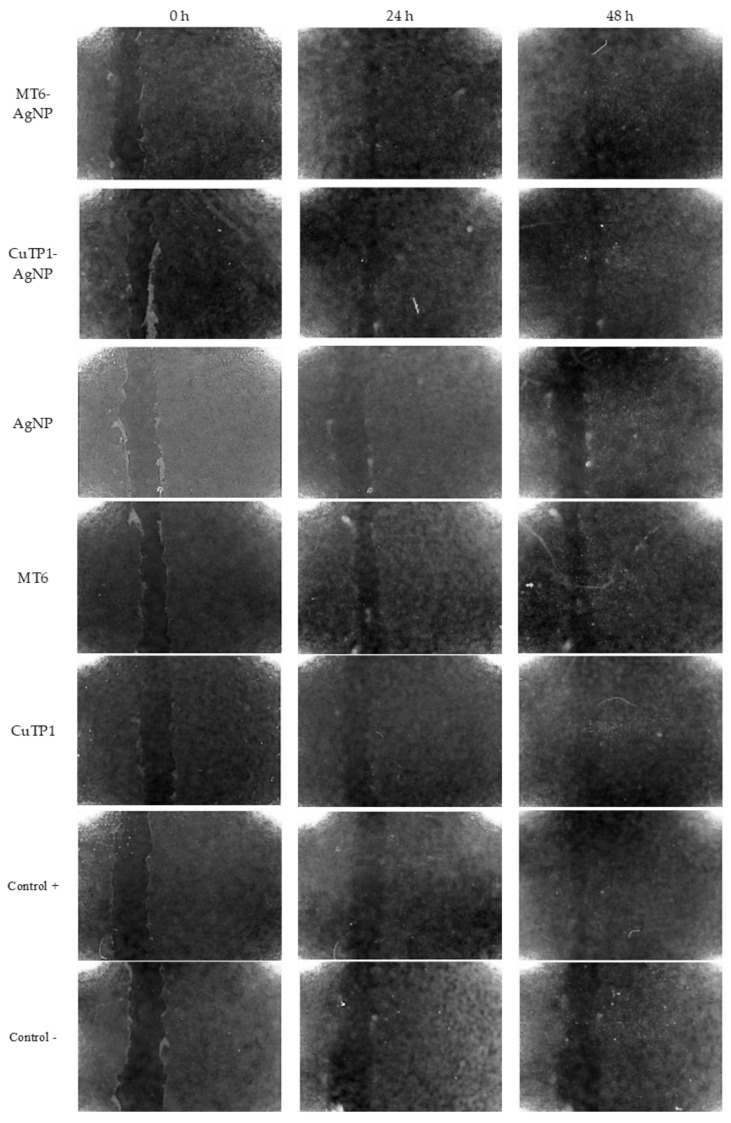
Microphotographs of the scratches prior and during the 48 h incubation of the NIH/3T3 cells with peptide–AgNP conjugates and the controls.

**Figure 5:**
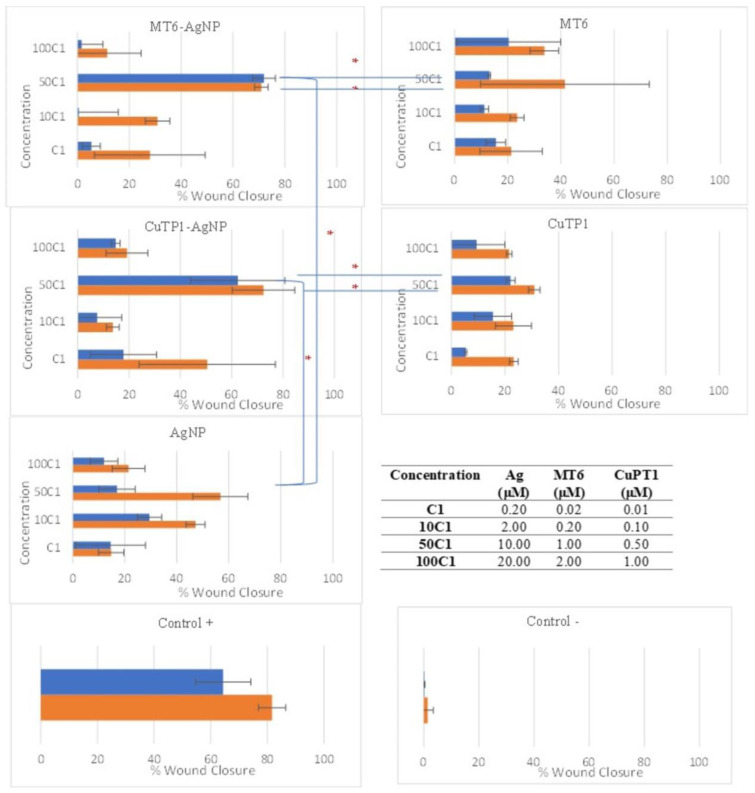
Scratch area reduction percentage (% wound closure) caused by incubation of fibroblasts with peptide–AgNP conjugates, the corresponding free peptide, conventional AgNP and positive (Control+) and negative (Control−) controls at 24 (■) and 48 (■) h. Statistical significance is denoted by * (*p* < 0.05).

The NIH/3T3 cells were enriched with different concentrations of peptide–AgNP conjugates (MT6-AgNP, CuTP1AgNP) and controls (corresponding free peptides MT6 and CuTP1, AgNP, Control+ and control−) and were incubated for 48 h at 37 °C (Figure 4). All peptide–AgNP conjugates and the free peptides exhibited significantly better scores at 50 C1 that corresponds to concentrations of 10 μM Ag for AgNP and 1 μM MT6 or 0.5 μM CuTP1 peptides. The results of the scratch area change over time are presented in Figure 5. 

After 24 h of incubation, MT6-AgNP exhibited 71.97 ± 4.35% reduction (*p* < 0.05) in the scratch area (wound closure) that remained stable at 48 h (70.92 ± 2.62%, *p* > 0.05). Incubation of the cells with MT6 peptide resulted in 13.13 ± 0.42% reduction at 24 h, about 5.48-fold lower than the corresponding MT6-AgNP conjugate (*p* < 0.05). At 48 h, this difference was reduced since the wound closure reached 41.48 ± 31.74% but remained still significantly lower (*p* < 0.05).

Similarly, CuTP1-AgNP exhibited 62.37 ± 18.33% wound closure at 24 h that remained at the same level at 48 h (72.31 ± 12.17%, *p* > 0.05). This score was also improved by 2.82-fold (*p* < 0.05) compared to the corresponding free CuTP1 (22.08 ± 1.68% at 24 h and 30.94 ± 2.15% at 48 h). Although the concentration of Ag needed for AgNP to exhibit the best wound closure was low (2 μΜ of Ag), the overall score at 24 h was much lower (*p* < 0.05) compared to either peptide–AgNP conjugate (29.53 ± 4.71% at 24 h and 47.26 ± 3.64% at 48 h). 

## 4. Discussion

In the present study, we studied the conditions of synthesis and its impact on the physicochemical properties of the peptide–AgNP that can enhance the migration rate of the fibroblast NIH/3T3 cell line across a scratch area. 

The peptides used in this work have successfully influenced the synthesis of peptide-AgNP conjugates with characteristics that ameliorate their wound healing potential. The success of the synthesis of silver nanoparticles was monitored using UV–vis spectroscopy, a method frequently used for the investigation of silver colloids due to its sensitivity in detecting the intense absorption peak corresponding to the surface plasmon excitation of silver nanoparticles. Depending on the particle size of silver nanoparticles, absorption bands between 350 and 450 nm appear, while they are approaching the red spectrum when the size increases [46]. The wavelengths of the maximum absorption of CuTP1-AgNP and MT6-AgNP were shifted with higher values (456 nm and 435 nm respectively) compared to AgNP that was synthesized without peptide interaction and that exhibited a band at 399 nm, in agreement with similarly synthesized silver nanoparticles [47]. The Miller indices and XRD spectra confirmed the formation of crystalline silver nanoparticles with a face-centered cubic lattice [48].

The mean particle size of CuTP1-AgNP and MT6-AgNP (181.600 ± 3.554 nm and 126.900 ± 3.754 nm respectively) was also significantly higher (*p* < 0.05) than AgNP (91.430 ± 0.658) that was similar with the values reported for silver nanoparticles synthetized using a similar method [49].

Τhe order in which the ingredients were added during the silver nanoparticle synthesis seems to influence the characteristics of the peptide–AgNP conjugates such as shape and size. The addition of the peptides prior to the reduction of AgNO_3_ has led to silver nanoparticles with larger hydrodynamic diameters compared to the ones that were formed by adding the peptide after the synthesis of nanoparticles. Additionally, the reaction’s yield was in favor of the first method, probably because the peptides could readily interact with the surface of nanoparticles, forming a denser capping as was revealed using electron microscopy. This capping halo was not observed on nanoparticles that interacted with the peptide after their formation (AgNP-MT6, AgNP-CuTP1). The higher yields of the peptide–AgNP conjugates that were synthesized in the presence of peptides (MT6 AgNP, CutP1 AgNP) compared to the ones in which peptides were added to the synthesized AgNP (AgNP MT6, AgNP CuTP1 respectively) is in accordance with the absorption intensities that are summarized in Table 1. The reason behind these results may be due to the fact that multiple functional moieties of the peptides like sulfhydryl, amino and carboxyl groups render their affinity to Ag atoms favorably as they can easily form chelated complexes, stabilizing the formed conjugates [33]. The effective capping of AgNP produced by the peptides during their synthesis is depicted in Figure 2. These results are verified by the particle size measurement. Image analysis of photographs obtained using TEM revealed similar distributions for all nanoparticles, as every particle was measured individually. DLS on the other hand presents results of the peptide–AgNP conjugates or even aggregates that may be present on each sample.

The negative ζ-potential values of all nanoparticles were attributed to the citrate stabilizer during the synthesis procedure [49]. Although the positive ζ-potential of the dispersion of silver nanoparticles is reported to exhibit an enhanced antibacterial efficiency due to the electrostatic interactions between the nanoparticles and the negative charges of the bacterial cell wall, this cytotoxic action is displayed mainly against mammalian cells and tissues [50,51]. This fact renders the positively charged particles more cytotoxic to the healthy tissue, compared to silver nanoparticles with neutral or negative charge [52]. Furthermore, ζ-potential with an absolute value above 15 mV indicates a satisfactory long-term stability of dispersions of nanoparticles that was confirmed by the stability study [51,53]. The higher negative values of ζ-potential of peptide–AgNP conjugates that were synthesized in the presence of the peptides (MT6-AgNP, CuTP1-AgNP) compared to the nanoparticles that have interacted with the peptide after their synthesis (AgNP-MT6, AgNP-CuTP1) indicate a stronger repulsion between the particles, a property that leads to enhanced long-term stability. Apart from ζ-potential that is influenced by the ionic strength and pH of the continuous phase, as well as the content of the peptides, several other parameters such as the structure organization of nanoparticles and their surface composition should be considered to sufficiently estimate their long-term stability [54]. The dense capping of MT6-AgNP and CuTP1-AgNP as depicted in TEM images (Figure 2) may also contribute to the stability of the conjugates.

Wound healing is a rather complicated process in which the migration of fibroblasts plays an important role [55]. Our results, for the very first time, demonstrated that both peptide–AgNP conjugates (MT6-AgNP, CuTP1-AgNP) can more prominently promote cell migration and therefore wound healing (*p* < 0.05) compared to the corresponding free peptides (MT6, CuTP1) or peptide-free AgNP. The wound healing effect was similar for both free peptides and did not differ significantly compared to the control+ (*p* > 0.05). The results demonstrated that the incubation of fibroblasts with peptides conjugated with silver nanoparticles showed a high migration rate, which is indicative of both the active proliferation and growth of the fibroblasts. This observation may stem from the expression of a contractile element in the fibroblasts such as myofibroblasts that may be the product of fibroblast differentiation caused by the application of silver nanoparticles [11]. Also, the enhanced performance of peptide–AgNP conjugates compared to the individual ingredients may be due to more efficient peptide targeting as peptide–AgNP conjugation may lead to the increase in local concentration of peptides [56]. Previous reports have emphasized that antimicrobial peptide–AgNP conjugates may serve as effective safe antimicrobial materials for wound treatment [57,58,59]. 

Various biomedical products are combined with silver nanoparticles and used to effectively resist or treat infections caused by various microbes and to expedite wound healing [12]. In addition, solutions with different concentrations of ionic silver compounds are used for wound treatment, commonly referred to as “colloidal silver” [60,61]. In order to effectively manage chronic wounds, wound care management mostly depends on the exploration of innovative and efficient wound dressing materials. There is, indeed, an increasing need for wound care, and proper wound care management is a significant clinical challenge. However, currently, there is a much better understanding of the pathogenesis of chronic wounds, and research and development of wound dressing materials have reached a new level of standard.

Finally, the main limitation of our study is that our findings are based on in vitro observations. Nevertheless, future studies will be required to test the hypothesis raised by our data before studies of efficacy are performed at a clinical level.

## 5. Conclusions

The present study focuses on the synthesis and characterization of peptides MT6 and CuTP1 conjugated with silver nanoparticles to explore their effect on skin-related wound healing process. It was found that the order in which the ingredients were added during the synthesis of nanoparticles influences the characteristics of the peptide–AgNP conjugates such as shape and size. Both peptide–AgNP conjugates that were tested more prominently promoted cell migration and therefore wound healing (*p* < 0.05), compared to the corresponding free peptides or peptide-free AgNP. The wound healing effect was similar for both peptides. Our results pave the way for more efficient wound treatment strategies as the new conjugates are promising ingredients for the development of innovative wound dressings and formulations for topical application.

## Figures and Tables

**Table 1:** Maximum absorption wavelengths of the synthesized silver nanoparticles.

Sample	Wavelength (nm)	Absorption
AgNP	399	1.079
MT6-AgNP	435	0.687
AgNP-MT6	415	0.433
CuTP1-AgNP	453	0.688
AgNP-CuTP1	397	0.303

**Table 2:** Yield of synthesis of silver nanoparticles and their physicochemical characteristics.

Sample	Ag Concentration of Dispersions of Silver Nanoparticles (ppm)		Yield (%)	Mean Size (nm)	PdI	ζ-Potential (mV)
	Theoretical	Measured				
AgNP	410.33 ± 1.11	363.88 ± 0.97	88.68 ± 0.20	60.44 ± 0.96	0.510 ± 0.010	−22.10 ± 1.48
MT6-AgNP	269.25 ± 5.06	222.61 ± 2.53	82.70 ± 1.81	126.90 ± 3.75	0.426 ± 0.004	−28.80 ± 0.46
AgNP-MT6	82.06 ± 1.76	46.50 ± 3.75	56.74 ± 5.69	91.43 ± 0.66	0.426 ± 0.020	−17.50 ± 0.55
CuTP1-AgNP	269.25 ± 5.42	174.41 ± 1.76	64.80 ± 1.58	181.60 ± 3.55	0.375 ± 0.017	−23.90 ± 1.21
AgNP-CuTP1	82.06 ± 2.01	10.78 ± 1.52	13.14 ± 2.01	122.30 ± 6.49	0.428 ± 0.054	−12.60 ± 1.84

**Table 3:** The average crystallite size (D) of silver nanoparticles calculated using the Scherrer formula.

Sample	2θ	FWHM ^1^	β_hkl_ ^2^	D (nm)
AgNP	38.13	0.52	0.00908	0.35
MT6-AgNP	θ°	0.04	0.00314	4.53
AgNP-MT6	19.065	0.18	0.00070	1.01
CuTP1-AgNP	$\cos\theta_{hkl}$	0.04	0.00070	4.53
AgNP-CuTP1	0.95	0.01	0.00018	18.13

^1^ Full width half maximum, ^2^ the half-width of the diffraction band.

**Table 4:** Particle size distribution, SAED patterns and Miller indices of the silver nanoparticles.

Sample	Particle Size Distribution	SAED Patterns	Miller Index			
			Ag (111)	Ag (220)	Ag (200)	Ag (311)
			d-Spacing (nm)			
AgNP	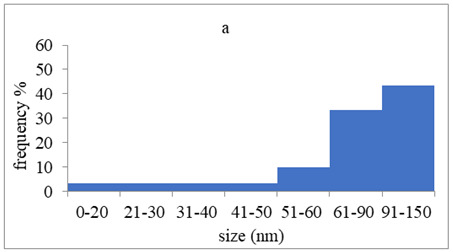	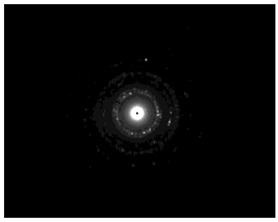	0.2403	0.2098	0.1458	0.1233
MT6-AgNP	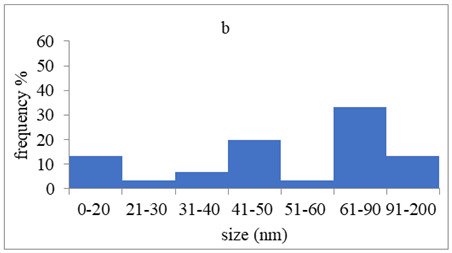	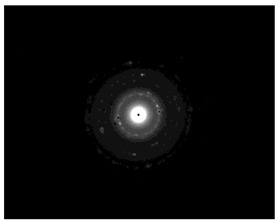	0.2386	-	0.1412	0.1241
AgNP-MT6	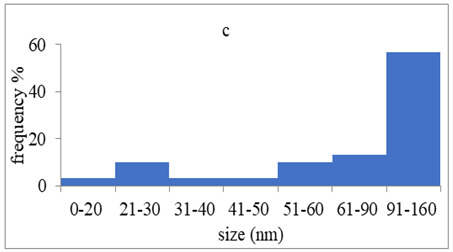	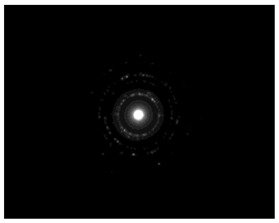	0.2391	0.2088	0.1458	0.1244
CuTP1-AgNP	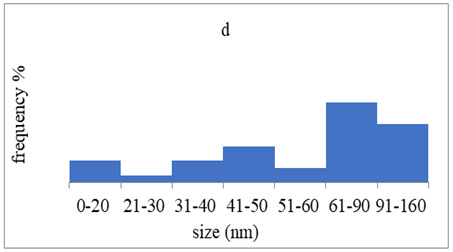	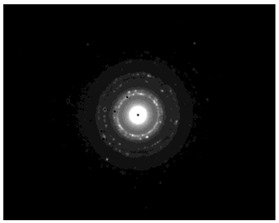	0.2412	0.2088	0.1463	0.1241
AgNP-CuTP1	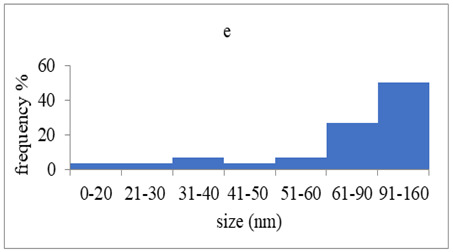	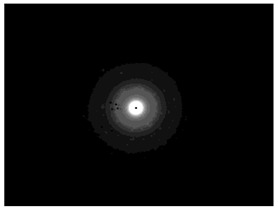	0.2413	0.2038	0.1449	0.1244

## Data Availability

Not applicable.
